# Serratus Anterior Muscle Injury in an Overhead-Throwing Athlete: A Report of a Rare Case With Serial Ultrasonographic Observations

**DOI:** 10.7759/cureus.92436

**Published:** 2025-09-16

**Authors:** Ryo Futamura, Masashi Kawabata, Hiroki Nakagawa, Hidenori Futamura, Katsumasa Sugimoto

**Affiliations:** 1 Department of Rehabilitation, Nagoya Sports Clinic, Nagoya, JPN; 2 Department of Rehabilitation, Kitasato University School of Allied Health Sciences, Sagamihara, JPN; 3 Department of Orthopedics, Nagoya Sports Clinic, Nagoya, JPN

**Keywords:** baseball pitcher, overhead athlete, return to play, serratus anterior muscle injury, ultrasonography

## Abstract

Serratus anterior muscle injuries in overhead athletes are extremely rare, and no consensus exists regarding treatment strategies or return-to-play timelines. We report the case of a high school baseball pitcher who sustained a serratus anterior injury. Ultrasonography revealed swelling and a hypoechoic area in the lower fibers of the serratus anterior at the level of the seventh and eighth ribs. Based on these findings, conservative management with localized compression was initiated, without the use of any medications, including nonsteroidal anti-inflammatory drugs. Serial ultrasonographic monitoring confirmed resolution of the inflammatory findings, after which contraction and stretching exercises were introduced. A progressive increase in throwing intensity enabled a return to competition 32 days post-injury. No reinjury occurred, and competitive play was maintained for six months. This case demonstrates that ultrasonographic monitoring is useful for guiding the progression of conservative therapy and determining the appropriate timing for return to play in serratus anterior injuries, providing clinically relevant insight into this rare throwing-related shoulder pathology.

## Introduction

Although muscle injuries are relatively common in overhead athletes, injuries of the serratus anterior muscle are exceptionally rare [1]. During the throwing motion, particularly from the late cocking to the follow-through phases, forceful contraction of the serratus anterior due to trunk rotation and scapular protraction may induce fatigue and contribute to muscular injury [1,2]. Although a few case reports have been published in rowing athletes [2,3], serratus anterior injury associated with throwing remains extremely uncommon [1].

The serratus anterior originates from the first through ninth ribs and inserts along the anterior medial border of the scapula [2]. It plays a crucial role in scapular protraction, upward rotation, and stabilization against the thoracic wall. Dysfunction of this muscle can compromise normal scapulothoracic motion and contribute to shoulder pain or impaired overhead performance, highlighting the clinical importance of recognizing such injuries.

Conservative treatment is typically the first-line approach, focusing on tissue repair through rest from sports activities, immobilization, and administration of nonsteroidal anti-inflammatory drugs (NSAIDs) [2]. This is followed by stretching and progressive athletic rehabilitation [2]. In cases unresponsive to conservative management, or when accurate diagnosis or anatomical repair is necessary, surgical intervention may be considered [1].

Gaffney reported a case of a rower who returned to sport after four weeks of activity restriction and physiotherapy [3]. Otoshi et al. described an avulsion injury of the serratus anterior treated surgically, with return to competition seven months postoperatively [1]. Although both conservative and surgical treatments can facilitate return to sport, the rarity of this condition means that consensus on recovery timelines and treatment progression remains limited. To the best of our knowledge, there have been no reports of serratus anterior injuries managed conservatively with serial ultrasonographic assessments to monitor healing and guide rehabilitation.

Here, we report a case of serratus anterior injury in a high school baseball pitcher, in which rehabilitation was guided by serial ultrasonographic monitoring, resulting in a safe and timely return to sport.

## Case presentation

The patient was a 17-year-old left-handed male high school baseball pitcher. He experienced severe left-sided chest pain on the fourth consecutive day of pitching (approximately 100 throws per day), specifically during the late cocking to acceleration phases of the throwing motion. He presented to our clinic two days later and was diagnosed with an injury to the left serratus anterior muscle.

**Figure 1:**
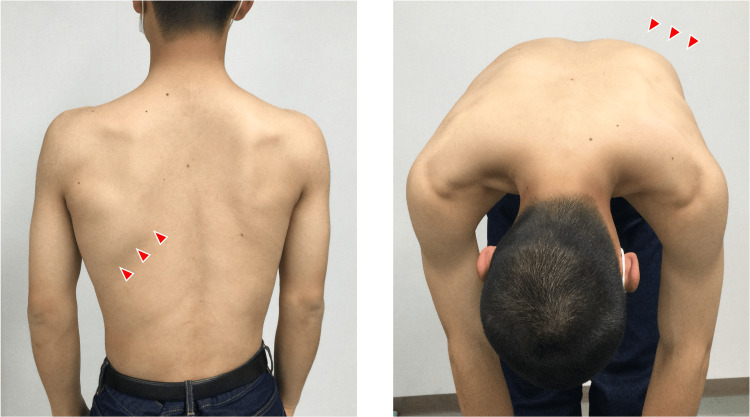
Visual inspection of the affected region Swelling and warmth were noted on the lateral chest wall, extending toward the posterior thorax at the level of the seventh and eighth ribs.

Initial evaluation revealed swelling and warmth over the lateral thorax at the level of the seventh and eighth ribs (Figure 1). The patient reported localized tenderness with a Numerical Rating Scale (NRS; 0 = no pain, 10 = worst possible pain) score of 9, pain with deep inspiration, and a sensation of tightness over the lateral thoracic wall. Active shoulder elevation was limited to 160°, with pain occurring above 100°. Pain during serratus anterior contraction was evaluated at shoulder flexion angles of 30°, 90°, and 120°, with reproduction of symptoms noted during scapular protraction at 120°. No scapular winging or tenderness was observed in the rotator cuff muscles.

**Figure 2:**
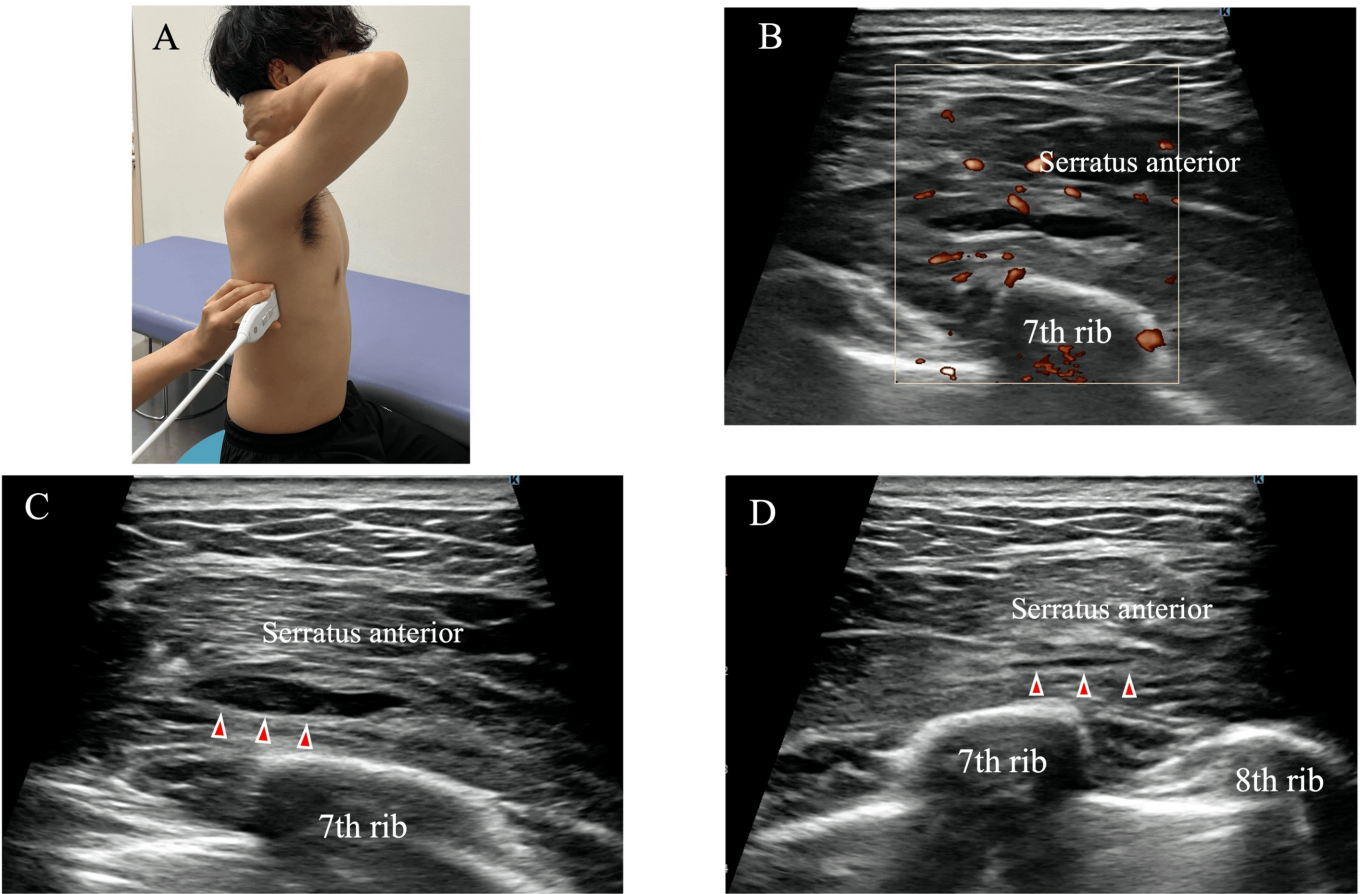
Ultrasonographic findings at the injury site (A) Patient positioned to visualize the lower fibers of the serratus anterior muscle. (B) Color Doppler imaging showing increased vascular signals around the hypoechoic area. (C) Hypoechoic area clearly visible without probe compression. (D) Hypoechoic area decreased in size under manual probe compression, suggesting a compressible lesion such as a hematoma.

**Figure 3:**
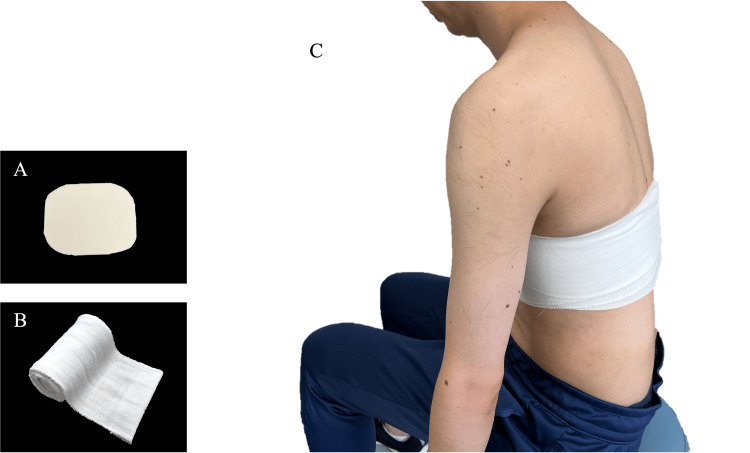
Application of local compression A soft pad was placed over the injury site, and localized compression was applied using an elastic bandage. (A) Soft pad used for local compression (YOU-Plast; Keiai Prosthetic Materials Sales Corp., Tokyo, Japan). (B) Elastic bandages used for local compression (READIFLEX A, GN403; Okamoto Industries, Inc., Tokyo, Japan). (C) Clinical appearance after local compression.

Ultrasonographic assessment demonstrated swelling of the lower fibers of the serratus anterior at the level of the seventh and eighth ribs, with a hypoechoic area approximately 2 cm in diameter and increased blood flow signals (Figure 2). The hypoechoic region was compressible under probe pressure (Figure 2), suggesting a localized hematoma. No medications, including NSAIDs, were prescribed during the treatment course; conservative management consisted solely of localized compression and rehabilitation exercises. Local compression was applied using a soft pad (YOU-Plast; Keiai Prosthetic Materials Sales Corp., Tokyo, Japan) and an elastic bandage (READIFLEX A, GN403; Okamoto Industries, Inc., Tokyo, Japan). The pressure was titrated to the minimal level that visibly reduced the hypoechoic area on ultrasound without causing patient discomfort, and no absolute pressure value was measured (Figure 3).

**Figure 4:**
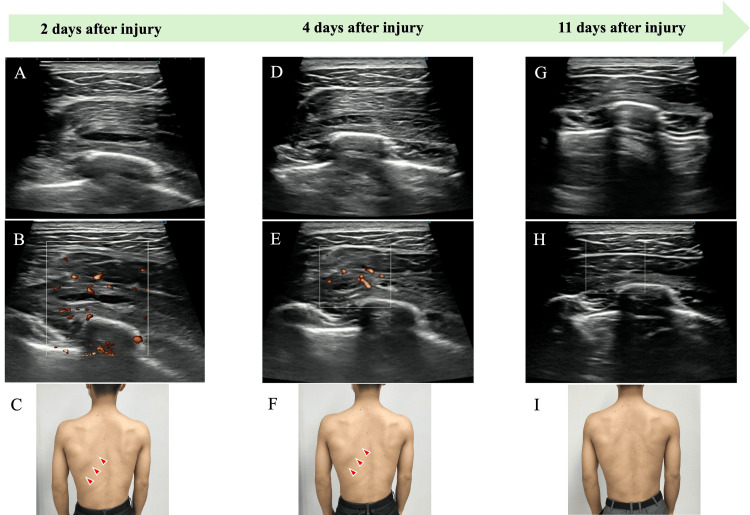
Serial ultrasonographic and clinical observations On days 2-4 post-injury, a hypoechoic area, increased blood flow signals, and localized swelling were observed. By day 11, the hypoechoic area and blood flow signals had resolved, and swelling had markedly decreased. (A) Ultrasound image two days after injury. (B) Color Doppler image two days after injury. (C) Clinical appearance two days after injury. (D) Ultrasound image four days after injury. (E) Color Doppler image four days after injury. (F) Clinical appearance four days after injury. (G) Ultrasound image 11 days after injury. (H) Color Doppler image 11 days after injury. (I) Clinical appearance 11 days after injury.

By day 11 post-injury, swelling and warmth had subsided (Figure 4). Ultrasonography confirmed resolution of the hypoechoic area and blood flow signals, after which local compression was discontinued. Although mild tenderness remained (NRS score, 3), both contraction- and stretching-induced pain in the serratus anterior had resolved. At this stage, active exercise was initiated to facilitate muscle contraction and stretching. In the side-lying position, the therapist supported the patient’s arm ventrally to allow controlled scapular protraction. Ultrasonographic monitoring confirmed no gapping at the injury site, and exercise intensity was maintained at a constant level. Home-based exercises included scapular protraction using a balance ball positioned anteriorly in the side-lying position to promote serratus anterior activation.

By day 18 post-injury, all symptoms had resolved, and the orthopedic physician in charge approved the resumption of throwing. Progressive concentric and eccentric training of the serratus anterior was introduced within pain-free limits, together with exercises to improve scapulohumeral and scapulothoracic mobility and stability. Ultrasonographic follow-up confirmed the absence of new hypoechoic regions or increased blood flow, supporting a progressive increase in throwing intensity. By day 32, the athlete had returned to competition under a 50-pitch limitation. Two months post-injury, there was no recurrence of symptoms, and the patient continued playing without reinjury six months later. A timeline of the clinical course, ultrasonographic findings, and interventions is provided in Table 1.

**Table 1:** Timeline of clinical findings, ultrasonographic observations, and interventions NRS, Numerical Rating Scale

Day (post-injury)	2-4	11	18	32	2-6 months
Clinical findings	Severe pain (NRS 9); swelling and tenderness at the seventh to eighth ribs; pain on deep inspiration; limited shoulder elevation; pain with scapular protraction; no scapular winging	Symptoms improved (NRS 3); swelling and warmth resolved; contraction- and stretching-induced pain resolved	All symptoms resolved; cleared to resume throwing	Returned to competition under a 50-pitch limitation; asymptomatic	Symptom-free; no reinjury during competitive play
Ultrasonographic findings	Hypoechoic area (~2 cm) in lower serratus anterior fibers; increased blood flow; compressible under probe	Resolution of hypoechoic area; vascular signals resolved	No new hypoechoic area; no vascular signals	Stable findings; no abnormalities	No abnormalities on follow-up ultrasound
Interventions	Local compression with a soft pad and elastic bandage; rest and ultrasound monitoring	Compression discontinued; initiation of stretching and activation exercises	Progressive concentric and eccentric serratus anterior training; scapulothoracic stabilization	Gradual increase in throwing intensity; supervised training	Full return to competition; continued monitoring

## Discussion

In this rare case of serratus anterior muscle injury, serial ultrasonographic monitoring enabled phase-specific rehabilitation and facilitated a return to competition by day 32 post-injury. This finding suggests that ultrasound imaging can serve as a valuable tool for clinical decision-making in muscle injury rehabilitation, including the timing of compression therapy, initiation of therapeutic exercises, and return-to-play clearance.

Muscle injuries are commonly assessed using both MRI and ultrasonography [4]. Although MRI provides high-resolution imaging of fine structural damage [4], it is time-consuming and less practical in acute sports settings. In contrast, ultrasonography is portable, immediate, and effective when combined with physical examination, offering prognostic information regarding return to play [5].

In the present patient, although serratus anterior injury was extremely rare, prompt ultrasonographic evaluation enabled a precise diagnosis and allowed therapy to progress in accordance with objective findings. We believe this approach contributed to an early return to sports without recurrence.

Anatomically, the serratus anterior originates from the first to ninth ribs and inserts at the superior, medial, and inferior angles of the scapula [6]. It consists of upper, middle, and lower fiber groups, with all reported sports-related injuries involving the lower fibers [1-3]. Similarly, in this patient, the injury was localized to the lower fibers at the seventh to eighth rib levels. The patient had engaged in high-volume throwing for three consecutive days prior to injury onset and reported shoulder tightness and difficulty elevating the arm. It is likely that continued throwing on the fourth day, despite these symptoms, led to the injury. Compensatory trunk and scapulothoracic motions due to shoulder dysfunction may also contribute to excessive loading on the serratus anterior [1].

While this case highlights the utility of serial ultrasonographic monitoring for guiding rehabilitation and return-to-play decisions, several limitations should be acknowledged. First, an MRI was not performed, which could have provided additional information for evaluating deep muscle injuries and differential diagnoses [7]. Second, objective measurement scales for muscle strength, range of motion, or throwing velocity were not collected, limiting the ability to quantitatively assess functional recovery. Finally, although the clinical features of this case were thoroughly documented, a standardized diagnostic checklist was not applied. Addressing these limitations in future reports, through comprehensive imaging, objective functional assessments, and standardized diagnostic criteria, could enhance reproducibility and clinical applicability.

## Conclusions

This case demonstrates that rehabilitation guided by serial ultrasonographic monitoring can facilitate a safe and timely return to sports in patients with serratus anterior muscle injury. Ultrasonography may serve as a practical and effective tool for planning treatment progression and determining return-to-play readiness following muscle injury. However, the absence of MRI evaluation and the single-patient nature of this report should be recognized as limitations, and further case reports are needed to confirm the generalizability of these findings.

## References

[1] Otoshi K, Itoh Y, Tsujino A, Hasegawa M, Kikuchi S (2007). Avulsion injury of the serratus anterior muscle in a high-school underhand pitcher: a case report. J Shoulder Elbow Surg.

[2] Carr JB 2nd, John QE, Rajadhyaksha E, Carson EW, Turney KL (2017). Traumatic avulsion of the serratus anterior muscle in a collegiate rower: a case report. Sports Health.

[3] Gaffney KM (1997). Avulsion injury of the serratus anterior: a case history. Clin J Sport Med.

[4] Crema MD, Yamada AF, Guermazi A, Roemer FW, Skaf AY (2015). Imaging techniques for muscle injury in sports medicine and clinical relevance. Curr Rev Musculoskelet Med.

[5] Woodhouse JB, McNally EG (2011). Ultrasound of skeletal muscle injury: an update. Semin Ultrasound CT MR.

[6] Nasu H, Yamaguchi K, Nimura A, Akita K (2012). An anatomic study of structure and innervation of the serratus anterior muscle. Surg Radiol Anat.

[7] Bordalo M, Arnaiz J, Yamashiro E, Al-Naimi MR (2023). Imaging of muscle injuries: MR imaging-ultrasound correlation. Magn Reson Imaging Clin N Am.

